# A Highly Efficient Graphene-Based Material for the Removal of Cationic Dyes from Aqueous Solutions

**DOI:** 10.3390/ma18040853

**Published:** 2025-02-15

**Authors:** Paunka Vassileva, Dimitrinka Voykova, Diana Kichukova, Tsvetomila Lazarova, Genoveva Atanasova, Daniela Kovacheva, Ivanka Spassova

**Affiliations:** Institute of General and Inorganic Chemistry, Bulgarian Academy of Sciences, Acad. G. Bontchev Str. Bl. 11, 1113 Sofia, Bulgaria; pnovachka@svr.igic.bas.bg (P.V.); dimitrinka@svr.igic.bas.bg (D.V.); kichukova@svr.igic.bas.bg (D.K.); lazarova@svr.igic.bas.bg (T.L.); genoveva@svr.igic.bas.bg (G.A.); didka@svr.igic.bas.bg (D.K.)

**Keywords:** graphene, toluidine blue, methyl violet, adsorption, dye removal

## Abstract

Graphene materials and their derivatives have shown promising capabilities in removing anionic and cationic dyes from wastewater. The present study aims at the synthesis of graphene-based material with a high specific surface area and evaluates its use as an adsorbent for removing toluidine blue and methyl violet from aqueous solutions. The physicochemical characterization of the adsorbent before and after dye adsorption is made by XRD, Raman spectroscopy, SEM, TEM, nitrogen physisorption, TG-DTA, and XPS. The influence of the solution’s pH, contact time, dye concentration, and temperature on the adsorption efficiency is investigated. The adsorbent demonstrated high adsorption capacity towards toluidine blue (265.2 mg.g^−1^) and methyl violet (200.4 mg.g^−1^) dyes from water. The adsorption process for both dyes follows the Langmuir model and involves physical rather than chemical interactions. Kinetic parameters were also determined. The adsorption of the studied cationic dyes can be attributed to a combination of mechanisms, including electrostatic interactions, hydrogen bonding, and π-π interactions between the dye molecules and the aromatic structure of reduced graphene oxide. The findings in the present work highlight the possibilities for enhancing graphene-based materials’ adsorption capabilities.

## 1. Introduction

Toluidine blue (TB) and methyl violet 6B (MV) are synthetic cationic dyes with diverse applications across various industries, including dyeing fabrics, coloring paper products, and use as components in the ink industry. Additionally, they are employed in biological and medical applications for staining tissues and cells [1]. However, both TB and MV are potentially carcinogenic and harmful to aquatic ecosystems [2,3]. Their presence in wastewater is associated with risks such as skin irritation, respiratory issues, and ecological disruption, making their removal from wastewater crucial [4,5].

**Figure 1:**
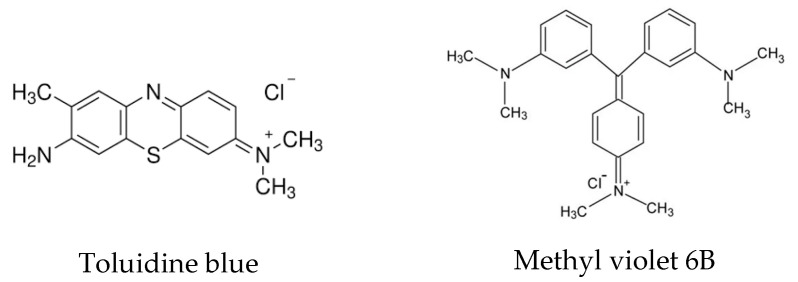
Structure of toluidine blue (CAS Nr. 92-31-9) and methyl violet (CAS Nr. 8004-87-3).

Adsorption has emerged as an effective and eco-friendly method for removing dyes like TB and MV from contaminated water. Structurally, toluidine blue is a thiazine dye characterized by a phenothiazine ring, while methyl violet is a triarylmethane dye consisting of a central carbon atom attached to three aromatic groups (Figure 1).

Despite these structural differences, both dyes exhibit similar adsorption mechanisms due to their positive charge and aromatic structure. Various materials, including natural and modified substances, have been identified as promising adsorbents for these dyes. A range of adsorbents, including agricultural waste-derived materials [6], natural zeolites [7], magnetic nanoparticles [8], alginate-based hydrogels [9], etc., have been effectively utilized for the removal of toluidine blue and methyl violet from water. A detailed comparison of the adsorption abilities of the most promising materials reported can be found further in Section 3. The large conjugated aromatic systems of the mentioned dyes facilitate interactions with adsorbents containing aromatic groups, such as activated carbon, biochar, and graphene-based materials [10].

Recently, some researchers have found that graphene nanomaterials and their derivatives show promising capabilities in environmental pollution control, such as heavy metals, anionic and cationic dyes, and gaseous emissions. Generally, the environmental applications of this type of material could be divided into three directions: adsorption, conversion, and detection. Graphene’s effectiveness in eliminating numerous pollutants is connected to its high specific surface area, the presence of various surface functional groups, and its fast electron transfer capabilities, which make it an excellent adsorbent, catalyst, and catalyst support. Due to its unique 2D structure, graphene can be a basic building block for graphitous materials of varying dimensions.

Graphene oxide (GO), a highly oxidized form of graphene, has garnered significant attention in recent years due to its exceptional electronic, thermal, optical, and mechanical properties [11,12]. GO contains oxygen-rich functional groups, including hydroxyl (-OH), epoxy (-O-), and carboxyl (-COOH), primarily located on its edges and surface [13]. These functional groups make GO hydrophilic and provide multiple adsorption sites for electrostatic interactions, hydrogen bonding, and π-π stacking [14,15]. Consequently, GO has shown strong potential as an adsorbent, with its high specific surface area, complex aromatic structure, amphiphilic nature, and oxygen-functionalized surface, enhancing its adsorption capacity, particularly for water treatment applications [11,16,17,18]. Because of the deprotonation of oxygen-containing groups (e.g., hydroxyl, carboxyl, and carbonyl) on graphene oxide nanosheets, the surface of GO is negatively charged [19,20]. GO facilitates strong electrostatic interactions with cationic dyes, which are positively charged in solution. This characteristic makes GO particularly effective for the adsorption of various cationic dyes from contaminated water, including methylene blue and crystal violet sources [11,14,21,22]. Additionally, its regenerative and reuse ability further enhances its potential for sustainable environmental remediation. Numerous studies have demonstrated GO’s effectiveness in adsorbing cationic dyes, making it a promising material for dye removal applications [23,24,25,26].

The goal of the present study is to apply an eco-friendly method for the synthesis of graphene oxide-based material with a high specific surface area and to evaluate its efficiency as an adsorbent for the removal of two representatives of cationic dyes—toluidine blue and methyl violet from aqueous solutions. This study also contributes to an in-depth investigation of the changes in the physicochemical characteristics of the adsorbent after the adsorption process, aiming to provide insight into the underlying mechanisms of the adsorption process.

## 2. Materials and Methods

### 2.1. Materials

The following compounds were used: synthetic graphite powder, H_2_SO_4_ (98%), KMnO_4_, H_3_PO_4_ (85%), toluidine blue (TB), and methyl violet (MV) dyes. All chemicals of analytical grade purity were from Merck KGaA, Darmstadt, Germany.

### 2.2. Preparation of Graphene Oxide

Graphene oxide was synthesized according to the eco-friendly method published by Marcano et al. [27]. The graphite powder was preliminary ground. Then, 1 g of graphite was placed in a beaker, followed by 40 mL of H_2_SO_4_ and 5 mL of H_3_PO_4_. The mixture was placed in an ice bath and stirred for 30 min. After that, 6 g of KMnO_4_ was dissolved in 100 mL of distilled water, and the solution was added dropwise. The resulting suspension was sonicated for 15 min and left to stand for 3 h. The sonicated mixture was filtered and washed with distilled water to pH 6.5. The prepared wet graphene oxide was lyophilized in a (ALPHA 1-2 LDplus, Christ GmbH, Osterode, Germany) freeze-dryer at 227 K and 0.057 mbar pressure for 24 h to obtain the final adsorbent. It was denoted as L-GO.

### 2.3. Characterization

Powder X-ray diffractograms were registered in a 5–80°2θ range with a Bruker D8-Advance Diffractometer (Karlsruhe, Germany) equipped with a Cu tube (λ = 1.5418Å) and a LynxEye detector. For the phase identification, EVA v4 software and the ICDD-PDF-2(2021) database were applied.

The morphology of the samples was examined using a combination of scanning electron microscopy (SEM) and transmission electron microscopy (TEM). SEM images were captured with a JEOL JSM-6390 (JEOL, Tokyo, Japan), while TEM analysis was performed on a JEOL 2100 microscope operating at 200 kV. The samples were placed on standard C/Cu grids for both techniques.

To analyze surface properties, N_2_ adsorption–desorption isotherms were obtained at 77 K using a Quantachrome Nova 1200e instrument (Anton Paar Quanta Tech Inc., Boynton Beach, FL, USA). The specific surface area was calculated via the BET equation, and the total pore volume and average pore diameter were derived from the adsorbed amount at a p/p_0_ near 1. Pore size distributions were determined using the NLDFT carbon equilibrium kernel based on the slit-pore model, while the Dubinin–Astakhov [28] method was used for micropore size distribution analysis.

The thermal analyses were performed in a LABSYSEvo (SETARAM, Calure, Paris, France) analyzer in a dynamic mode and argon flow in the range of 25–850 °C.

A Raman spectrometer LabRAM HR800 (HORIBA Jobin Yvon IBH Ltd., Glasgow, UK) with a 600 mm^−1^ diffraction grid equipped with an optical microscope was used. The wavelength was 633 nm (He-Ne laser) with a power of 40 μW; a ×100 objective was used to focus the laser.

FTIR analyses were carried out in a Nicolet iS5 spectrometer (Thermo Fisher Scientific, Waltham, MA, USA) with a resolution of 2 cm^−1^ in KBr.

X-ray photoelectron spectra (XPS) were collected using an ultra-high vacuum chamber of an ESCALAB-Mk II (VG Scientific, Manchester, UK) electron spectrometer with Al K_α1,2_ (hν = 1486.6 eV). The composition and bonding of the samples were investigated based on the areas and binding energies of the photoelectron peaks and Scofield’s photoionization cross-sections. XPSPEAK41 software was used to deconvolute the XPS data.

### 2.4. Adsorption Experiments

Stock solutions of toluidine blue and methyl violet dyes were prepared at a concentration of 1000 mg L^−1^ by dissolving the appropriate amount of dye powder in deionized water. All working solutions with the desired concentrations were then obtained by diluting the stock solution with deionized water.

The adsorption experiments were performed in 50 mL flasks. Initially, a measured quantity of a solid sample was added to 10 mL of a dye solution prepared with concentrations ranging from 50 to 1000 mg/L. These mixtures were then agitated on a rotary shaker for various durations. After agitation, the precipitate was removed from the solution using centrifugation. To analyze the dye concentrations, a spectrophotometer Vis DLAB SP-V1000 (DLAB Scientific Malaysia SDN BHD, Selangor, Malaysia) was used, measuring absorbance at λ_max_ = 582 nm for methyl violet and λ_max_ = 626 nm for toluidine blue. The calibration curves for both dyes are presented in Figures S1 and S2 in the Supplementary Materials. The batch adsorption experiments aimed to investigate the influence of contact time, pH, initial dye concentration, and temperature on the adsorption process.

The adsorption capacity of the dyes was calculated by the formula:

(1) $$Q_{e}=\left(C_{0}-C_{e}\right)\times V/m$$

where *C*_0_ and *C_e_* represent the concentrations of the initial and equilibrium dye solutions (mg/L), respectively, *V* is the applied volume of the solution (L), and *m* denotes the mass of the sorbent (g). Each experiment was performed three times to ensure precision and consistency.

After the adsorption experiments with toluidine blue and methyl violet dyes, the samples were dried and subjected to some physicochemical analyses to follow the adsorbent changes during this process. These samples are denoted as L-GO/TB and L-GO/MV.

## 3. Results and Discussion

### 3.1. Characterization of the Prepared Adsorbent

**Figure 2:**
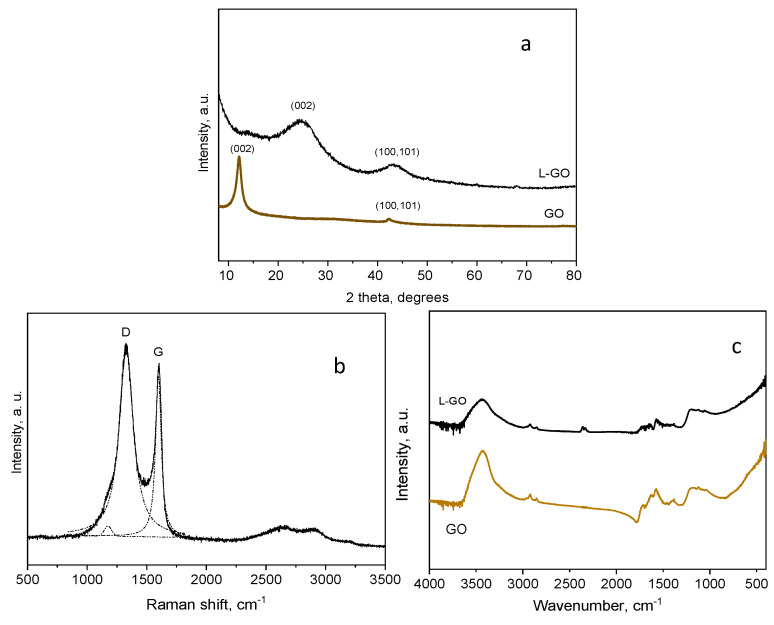
XRD (**a**), Raman spectrum (**b**), and FTIR spectra (**c**) of the as-prepared GO (brown line) and freeze-dried L-GO (black). Dotted lines in Raman denote deconvolution of the peaks.

XRD diffraction patterns of the graphene oxide before and after lyophilization are shown in Figure 2a. The pattern of the as-obtained GO contains a peak at 11.9°2θ, corresponding to the increased distance between the graphene sheets. The peak at 42.3°2θ originates from atoms ordering within the sheet. After the freeze-drying process, only broad humps are registered at about 24.7°2θ and at 42.5°2θ, which correspond to the (002) and (100, 101) peaks, respectively, of partially reduced graphene oxide. The width of the peak indicates the small crystallite size of the obtained material, which implies that the freeze-drying results in the partial evolution of functional groups containing oxygen from the graphene sheets. Thus, the lyophilized material is reduced graphene oxide rather than graphene oxide.

The Raman spectrum of L-GO is presented in Figure 2b. It consists of two intensive bands in the range of 1100–1700 cm^−1^. The D-band is registered at 1325 cm^−1^ and is associated with the disorder within the graphene sheet’s structure, as well as with the presence of a large number of edges due to the small sizes of the sheets (as seen from XRD). The intensity of this band is high for amorphous carbon and very weak in well-crystalline graphite. The G-band appears at 1607 cm^−1^. Its intensity correlates with the ring-stretching vibrations of sp^2^ C–C. The I_D_/I_G_ ratio between the intensities of the characteristic D and G peaks is often given as a measure of the defectiveness of the graphene structure. The I_D_/I_G_ = 1.2 in the case of L-GO also reflects the low average size of the sp^2^ domains. Additionally, low intensive bands at the 2500–3000 cm^−1^ region are observed and related to the 2D-overtone and D + G combinatorial band. The FTIR GO spectrum (Figure 2c) reveals a broad peak at 3434 cm^−1^, assigned to the O-H stretching vibration and the water content of the as-prepared graphene oxide. The peak at 1723 cm^−1^ corresponds to C=O stretching, while C-OH bending is observed at 1587 cm^−1^, and the peak at 1234 cm^−1^ is identified as C-O-C bending. The spectrum of the L-GO sample presents a band at about 3430 cm^−1^ due to OH- groups and water but with lower intensity. A similar decrease in the peaks’ intensities is observed for the carbon–oxygen groups at 1720 and 1577 cm^−1^, revealing their partial removal during the freeze-drying. The peak at 1630 cm^−1^ for C=C stretching appears in both spectra. The FTIR spectra confirm the partial reduction of GO upon freeze-drying registered by XRD analysis.

**Figure 3:**
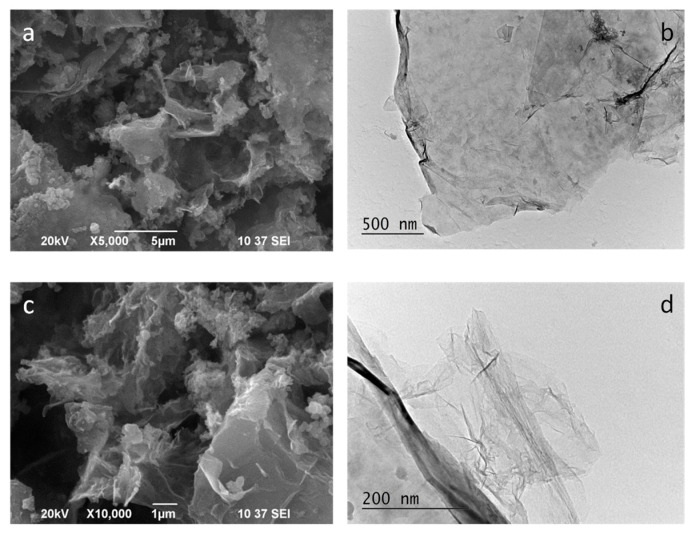
SEM (**a**,**c**) and TEM pictures (**b**,**d**) of L-GO.

A combination of SEM and TEM was used to investigate the morphology of the prepared L-GO material. The micrographs of the material with different magnifications are presented in Figure 3. The lyophilized material consists of randomly stacked, well-defined, unfolded graphene sheets with torn and wrinkled edges.

Such morphology is a prerequisite for the formation of a large number of active sites available on the surface for adsorption. The EDS analysis of the carbon-to-oxygen ratio, found to be equal to 87:13, reveals the partial reduction of the graphene oxide upon freeze-drying.

**Figure 4:**
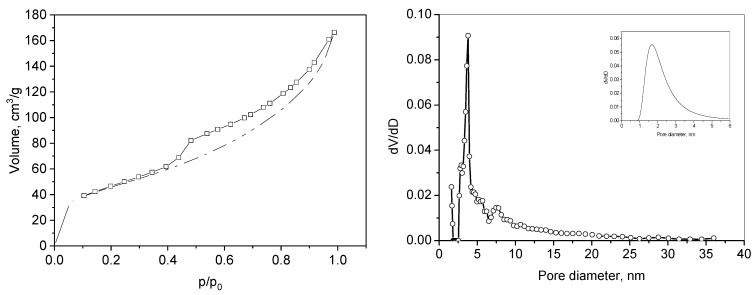
Nitrogen adsorption–desorption isotherm (**left**) and pore/micropore size distribution (**right**) of L-GO. Full symbols-adsorption, empty symbols-desorption.

The nitrogen isotherm of adsorption–desorption on L-GO and the micropore size distribution, according to the DA method [28], are presented in Figure 4. The specific surface area, total pore volume average pore size, volume of micropores (calculated by the V-t method), and mean diameter of the micropores are shown in Table 1. The isotherm, according to IUPAC classification, can be assigned to pseudo-type II, which appears with materials with slit-shaped pores or aggregates of platy particles [29].

This type of isotherm is not completely reversible, and the desorption branch can be registered, usually confined to the multilayer region. A hysteresis H3 is observed, evidencing pores with slit shapes formed from the voids between the plate-like layers of L-GO, and this type of hysteresis appears when the particles are non-rigid. The bimodal pore size distribution and a certain volume of micropores centered at 1.6 nm (V_mi_, Figure 3b) reveal some texture hierarchy of the prepared L-GO.

It should be noted that the freeze-dried L-GO sample possesses a high specific surface area and relatively high pore volume. A notable amount of micropores is determined. Hence, the applied procedure restricts the capillary forces and prevents the graphene sheets from sticking.

**Figure 5:**
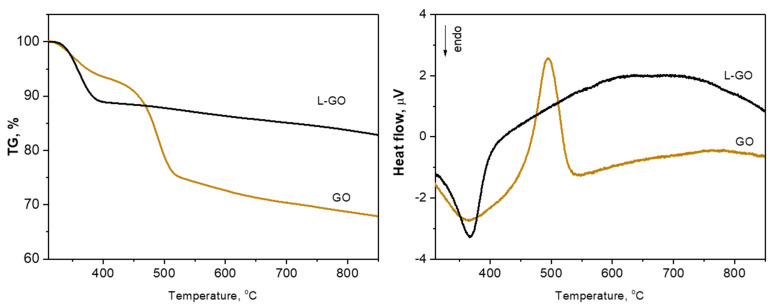
TG (**left**) and DTA (**right**) of the as-prepared GO and freeze-dried L-GO.

The TG/DTA curves of the as-prepared GO and the freeze-dried L-GO are presented in Figure 5. The TG curve of GO exhibits a typical three-step mass loss of graphene oxide in a range of 308–873 K. The registered total mass loss is 32.6% up to 873 K. The mass loss of the first step below 373 K of 7.7% is due to the release of the adsorbed water between the graphene layers [30,31]. The mass loss at the second step in the 423–548 K range of about 18.3% is attributed to the reduction of the various labile oxygen-containing functional groups (carboxylic, anhydride, or lactone) [32]. In the third step, at 523 K to 873 K, a gradual weight loss reflecting the removal of other stable oxygen formations (for instance phenol, carbonyl, quinone) of GO is registered [32].

The DTA curve of the material presents a strong exothermal peak centered at 495 K that reveals the reduction process of GO to RGO. The thermal profile of L-GO differs substantially from that of the as-prepared one. It consists of only one stage of weight loss (10%) up to about 373 K due to the evolution of the residual water after the freeze-drying process. The sharp endo-effect in the DTA curve confirms this process. Above this temperature, a gradual weight loss up to the final temperature with a residue of 85% is registered.

Thus, the thermal analyses also confirm that the freeze-drying process leads to a partial reduction of the as-prepared GO to reduced graphene oxide.

**Figure 6:**
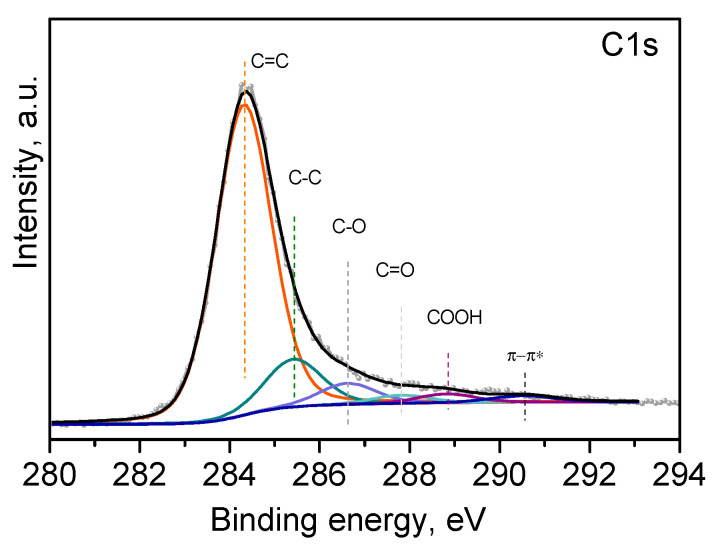
C1s spectrum of L-GO.

The surface state of the prepared material is evaluated by XPS analysis. Figure 6 presents the C1s spectrum of the freeze-dried sample, typical for reduced graphene oxide. The spectrum line is asymmetric towards the higher binding energies, which is related to the presence of different types of carbon bonds.

The following contributions that correspond to several carbon bonds, essential for RGO materials, are obtained after deconvolution of the experimental line: C=C bond (sp^2^) at 284.3 eV, C−C bond (sp^3^) at 285.4 eV, C−O (or epoxy) at 286.6 eV, C=O at 287.7 eV, COOH bond at 288.8 eV, and π-π* at 290.5 eV [33]. The chemical composition of the surface is 93% carbon and 7% oxygen, which is in good concordance with the data from EDS.

The XPS analysis of L-GO confirms the reduction of GO to RGO during the freeze-drying process.

All analyses unequivocally corroborate the partial reduction of the initially prepared GO to RGO. A similar effect of the partial reduction of GO to RGO upon freeze-drying was reported [34]. Another research study indicated that freeze-drying can create a three-dimensional porous architecture from graphene oxide hydrogels, which can result in the formation of graphitic microcrystals, suggesting a chemical reduction of the graphene oxide during the freeze-drying process [35].

### 3.2. Adsorption Studies on L-GO

This study demonstrates that the behavior of the obtained material in the adsorption of toluidine blue and methyl violet dyes from water solutions is significantly influenced by several factors, including pH, contact time, and initial dye concentration.

#### 3.2.1. Effect of pH

**Figure 7:**
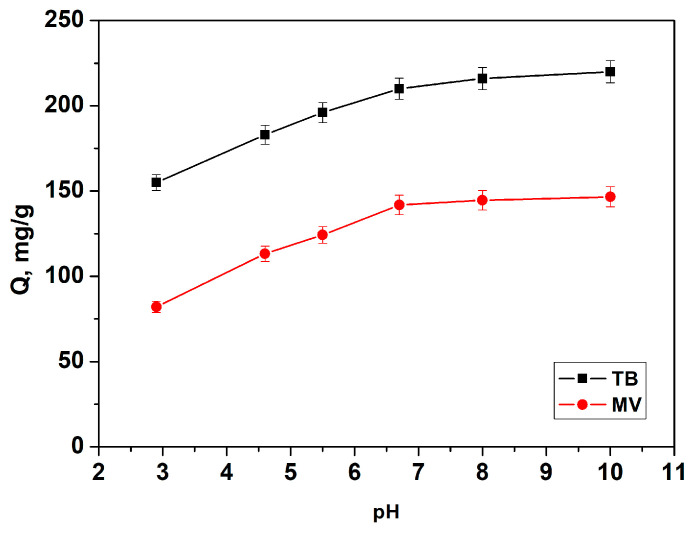
Effect of pH on dye adsorption by L-GO: initial dye concentration of 300 mg/L, adsorbent dose of 1 mg/L, contact time of 24 h, and temperature of 20 °C.

The initial pH of the dye solution plays a crucial role in determining dye adsorption capacity, as it influences both the ionization state of dye molecules and the surface charge of the adsorbent. Adsorption tests were conducted at pH values ranging from 2.8 to 10.0 with an initial concentration of 300 mg/L for both TB and MV, as shown in Figure 7. Within this range, both toluidine blue and methyl violet remain in their cationic forms, ensuring favorable electrostatic interactions with negatively charged adsorbent surfaces at higher pH levels. The results indicate that the adsorbed amount of both dyes increases steadily with rising pH, reaching a maximum at pH 10.0.

At low pH levels, the adsorbent surface is positively charged due to the protonation of functional groups, leading to electrostatic repulsion with the cationic dye molecules and lower adsorption. As the pH increases, functional groups, like -COOH and -OH on the adsorbent, deprotonate, generating negative charges that enhance electrostatic attraction, thereby improving dye removal [36,37]. The smaller increase in adsorption for TB and MV between pH = 7 and 10 suggests that mechanisms other than electrostatic attraction, such as hydrogen bonding, may also contribute to the adsorption process [38].

Although maximum adsorption was observed at pH = 10, all subsequent experiments were conducted at a neutral pH of 7.0. Additionally, according to Bonetto et al. [39], at pH values above 9.0, MV reacts with OH^–^ ions to form a carbinol base with altered molecular geometry, shifting the absorption peak to the UV region. This shift complicates adsorption studies by altering the dye’s absorbance profile.

#### 3.2.2. Influence of the Contact Time and Adsorption Kinetics

**Figure 8:**
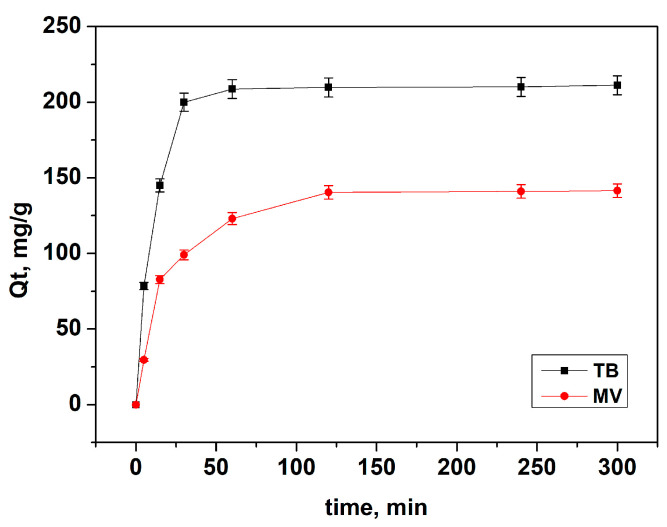
Influence of the contact time on the dye adsorption by L-GO: initial dye concentration of 300 mg/L, adsorbent dose of 1 mg/L, pH = 7, and temperature of 20 °C.

The influence of the contact time was examined at pH 7.0 with an initial dye concentration of 300 mg L^−1^ for both TB and MV. Figure 8 illustrates how the adsorbed amounts change over time.

The results show that the amount of both dyes adsorbed increased rapidly with longer agitation, reaching equilibrium at 60 min for TB and 120 min for MV. The longer time needed for MV to reach equilibrium is attributed to its larger size compared to TB, causing steric hindrance and slower diffusion. Additionally, the presence of multiple methyl groups and larger aromatic structures in MV enhances hydrophobic interactions and steric effects during the adsorption process.

Many studies have also demonstrated rapid adsorption rates of various dyes on GO and RGO [22,40]. This rapid kinetics is particularly advantageous in industrial settings, where time efficiency is essential.

To comprehend the adsorption mechanism, three kinetic models were utilized on the experimental data: the pseudo-first-order, pseudo-second-order, and intraparticle diffusion models. The specified models are expressed by the following equations:

1. Pseudo-first-order model:


(2) $$\log\left(Q_{e}-Q_{t}\right)=\log\left(Q_{e}\right)-\left(k_{1}/2.303\right)t$$

2. Pseudo-second-order model:


(3) $$t/Q_{t}=\left(1/k_{2}Q_{e}\right)+\left(1/Q_{e}\right)t$$

3. Intraparticle diffusion model:


(4) $$Q_{t}=k_{id}t^{1/2}+C$$

In these equations, Q_e_ and Q_t_ represent the amounts of dye adsorbed at equilibrium and at time t, respectively, while k_1_, k_2_, and k_id_ are the rate constants specific to each model. Additionally, C is a constant associated with the boundary layer thickness.

The calculated rate constants and correlation coefficients (r^2^) are presented in Table 2. A comparison of the pseudo-first-order and pseudo-second-order kinetic models reveals that the adsorption of both dyes onto the tested adsorbent aligns more closely with the pseudo-second-order kinetics. This finding suggests that chemisorption may be the rate-limiting step in the adsorption systems studied [41,42,43]. The sorption capacity for both cationic dyes appears to depend on the number of active sites occupied on L-GO [44]. Furthermore, the calculated rate constant k_2_ from this model confirms that MV adsorbs at a slower rate compared to TB.

The analysis of the intraparticle diffusion model indicates that the adsorption process for TB and MV occurs in multiple stages. The kinetic plots display two distinct linear regions, revealing a complex adsorption mechanism. The first linear segment corresponds to the rapid adsorption phase (denoted as k_id1_), influenced by boundary layer diffusion. The second linear segment (k_id2_) represents a slower adsorption phase, attributed to intraparticle diffusion, which acts as the rate-limiting step. The values of k_id2_ are lower than those of k_id1_, indicating slower diffusion in the second stage compared to the film diffusion rates in the first. Furthermore, the higher k_id_ values in both stages for the TB dye suggest a faster diffusion of this dye into the pores of the adsorbent compared to the MV dye.

#### 3.2.3. Adsorption Isotherms

Adsorption isotherms describe the relationship between the concentration of adsorbate in solution and the amount adsorbed on a surface at equilibrium. For the adsorption of TB and MV, three common models are typically used to provide insights into their adsorption behavior:

- The Langmuir isotherm models monolayer adsorption on a uniform surface with a finite number of identical sites available for adsorption. It is particularly useful for describing the maximum adsorption capacity, where each site can hold only one molecule, and no further adsorption can occur once all sites are occupied.
- The Freundlich isotherm is an empirical model that describes adsorption on heterogeneous surfaces with varying affinities, indicating multilayer adsorption.
- The Dubinin–Radushkevich (D-R) isotherm focuses on the adsorption process on microporous materials and helps determine the nature of adsorption (physical or chemical) based on the mean adsorption energy.

**Figure 9:**
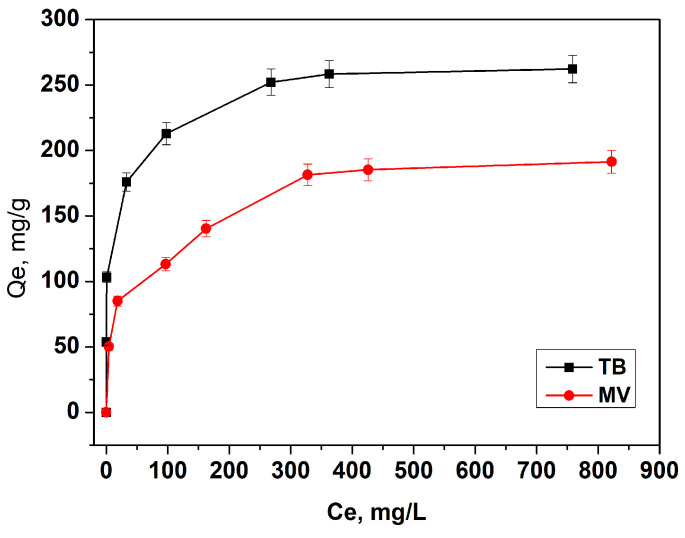
Influence of concentration on dye adsorption by L-GO: adsorbent dose of 1 mg/L, contact time of 5 h, and temperature of 20 °C.

Figure 9 and Table 3 present the experimental isotherms and the calculated values for the model isotherm constants, along with the correlation coefficients for both dyes. The adsorption data of TB and MV on L-GO showed an excellent fit with the Langmuir model, as supposed by the high correlation coefficients (r^2^ > 0.992). These results indicate that the dominant adsorption mechanism for both dyes involves the interaction with a relatively uniform surface and the formation of a monolayer. The maximum Langmuir capacity for TB is higher than that for MV. Toluidine blue adsorbs more effectively on the negative surface of L-GO for several reasons: TB has a smaller molecular size and carries a single, well-distributed positive charge, causing stronger electrostatic attractions with the negatively charged groups of L-GO. Additionally, due to its simpler structure and smaller size, TB can more easily access adsorption sites without being hindered by steric effects.

The n-values from the Freundlich isotherm for TB (5.52) and MV (3.82) indicate the favorability of adsorption onto L-GO [11]. The adsorption energy (E) from the D-R isotherm for the TB dye, E = 2.92 kJ/mol, suggests physisorption, as adsorption energies below 20 kJ/mol typically indicate physical adsorption. This implies weak interactions, likely involving electrostatic forces and van der Waals interactions. For the MV dye, E = 0.35 kJ/mol is even lower, pointing to a very weak physical adsorption process, likely dominated by electrostatic and dispersive forces. In both cases, the low E-values confirm that the adsorption is not chemisorption (which usually involves energies above 40 kJ/mol) but is instead physical in nature.

Notably, the comparison of the adsorption capacities for TB and MV shows that L-GO exhibits a higher adsorption capacity for both dyes compared to many other reported adsorbents, underscoring its suitability for cationic dye removal from aqueous solutions (Table 4).

#### 3.2.4. Effect of Temperature

**Figure 10:**
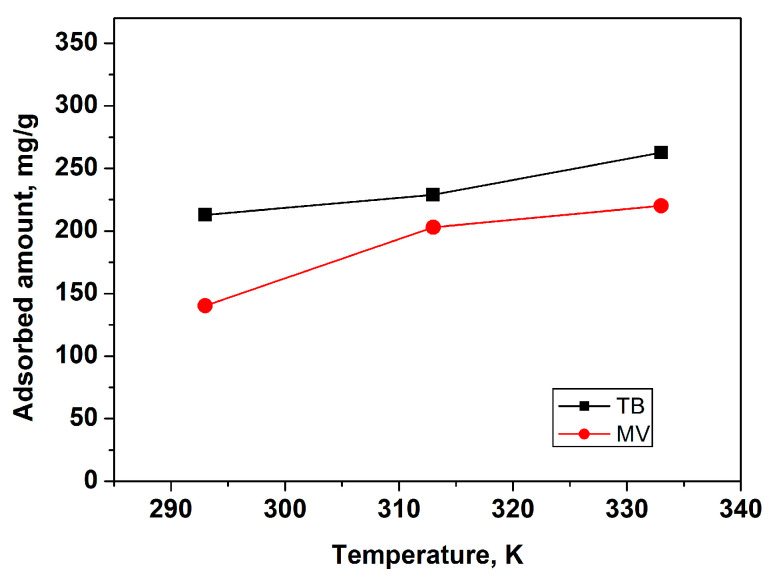
Effect of temperature for TB and MV adsorption: initial dye concentration of 300 mg/L, adsorbent dose of 1 mg/L, pH 7, and contact time of 5 h.

Temperature typically influences adsorption capacity and efficiency. The adsorption of TB and MV was investigated at three temperatures: 293, 313, and 333 K. As shown in Figure 10, the adsorbed amounts of both dyes increased with rising temperature, suggesting the endothermic nature of the adsorption process. Higher temperatures provide increased energy for the interaction between the molecules of the dyes and L-GO, leading to enhanced adsorption capacity and efficiency.

#### 3.2.5. Desorption

The regeneration of spent adsorbent is critical in dye adsorption studies. Desorption studies focus on releasing adsorbed dye molecules from adsorbent surfaces to assess the reversibility and stability of the adsorption process, which aids in evaluating adsorbent efficiency and reuse potential for water treatment.

Desorption experiments were conducted in batch mode to test L-GO regeneration after adsorbing the TB and MV dyes, using three desorbing agents: pure ethanol, a mixture of ethanol and 1M HCl (50:50), and a mixture of ethanol and 1M NaCl (50:50). The results are summarized in Table 5. Desorption percentages for both the TB and MV dyes follow similar trends across all desorbing agents, indicating that both dyes have comparable adsorption affinity on L-GO. The highest desorption percentage for both dyes was achieved with the ethanol and 1M NaCl mixture. The presence of NaCl likely promotes ion exchange between sodium ions and the cationic dye molecules, enhancing the desorption process [48]. The high desorption efficiency suggests that the adsorption of both dyes is primarily physical, consistent with previous findings.

### 3.3. Studies on L-GO After Toluidine Blue and Methyl Violet Adsorption

To shed light on the interactions of the adsorbent with the dyes, some physicochemical investigations were carried out. The L-GO samples after the respective dye adsorption are denoted as L-GO/TB and L-GO/MV.

**Figure 11:**
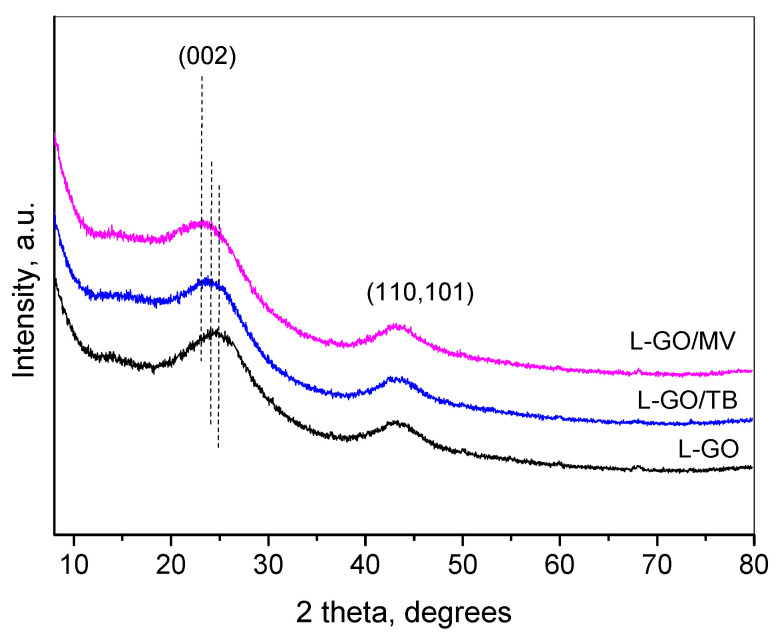
XRD of L-GO, L-GO/TB, and L-GO/MV. Dotted lines denote the maxima of the peak.

Figure 11 presents the XRD patterns for the material after TB and MV dye adsorption. The pattern of the initial adsorbent L-GO is shown for comparison.

The general view of the patterns after adsorption preserves the view of the initial one. A small shift in the maximum associated with the (002) interplanar distance toward higher values is observed, being more pronounced for the L-GO/MV sample. Such a shift may be due to the interaction of the graphene sheets with the molecules of the dyes. It should be noted that the position of the second hump at 42.5°2θ remains unchanged after adsorption, thus pointing that the distances within the graphene sheet are preserved.

**Figure 12:**
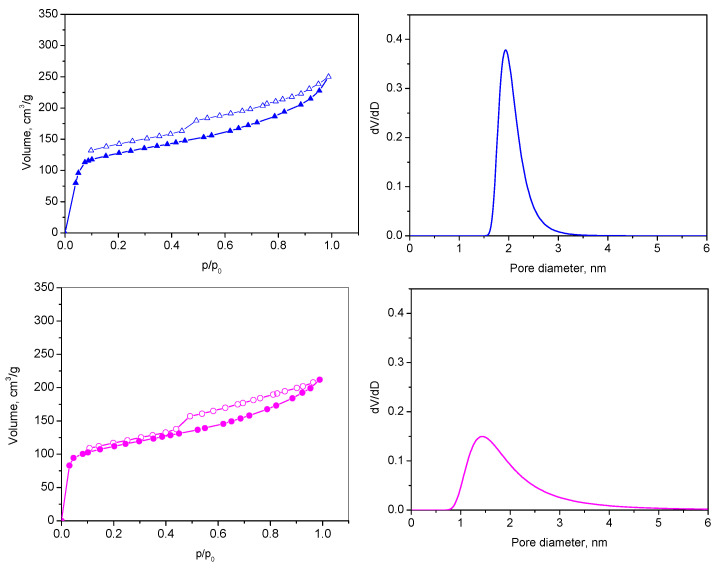
Nitrogen adsorption–desorption isotherm (**left**) and micropore size distribution (**right**) of L-GO/TB (blue) and L-GO/MV (magenta). Full symbols denote adsorption, empty symbols-desorption.

The values of the specific surface area and total pore volumes after the adsorption of the toluidine blue and methyl violet dyes of L-GO/TB and L-GO/MV in Table 6 are expected, as they reflect the partial filling or blocking of the pores with the dye molecules. The adsorption of both dyes does not change the type of isotherms and hysteresis loop (pseudo II type, H3), implying the preservation of the sheet-like structure of the adsorbent (see Figure 4 and Figure 12). The lowering of the texture values is more pronounced for L-GO/MV as the size of the molecule of methyl violet is larger. The volumes of the micropores also reflect this occasion and suggest that dye adsorption predominantly proceeds in this type of pore.

**Figure 13:**
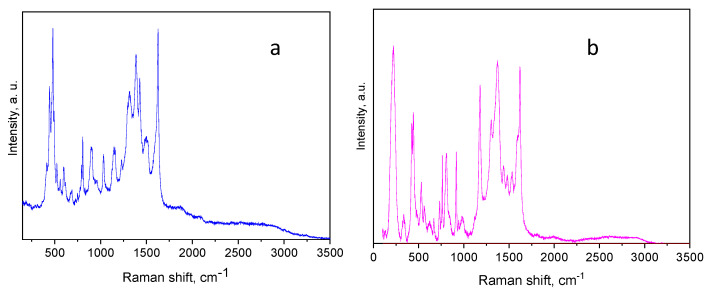
Raman spectra of L-GO/TB (**a**) and L-GO/MV (**b**).

The Raman spectra of L-GO/TB and L-GO/MV are presented in Figure 13.

The spectral data of L-GO/TB and L-GO/MV align closely with those for toluidine blue and methyl violet dyes, as documented in the literature [61,62]. Some prominent Raman bands, which appear in the range of 1625 to 1387 cm^−1^, are associated with the stretching vibrations of the TB skeleton. Bands registered at frequencies of 1147 cm^−1^ and 1028 cm^−1^ correspond to in-plane C–H bending vibrations. A strong band at 1387 cm^−1^ is assigned to C–C ring vibrations or C–N ring vibrations [63,64]. A combined stretching vibration of C-C and C-N is also envisaged for this band [65]. The peak observed at about 685 cm^−1^ is attributed to the breathing vibration of the ring, along with C–S and C–C stretching, while the stretching of C–S and C–C induce the bands in the range of 446 to 480 cm^−1^. Several key peaks may be mentioned when interpreting the L-GO/MV spectrum. The 1620 cm^−1^ and 1590 cm^−1^ peaks represent symmetric and asymmetric benzene modes, respectively. The peak at 1366 cm^−1^ is assigned to the stretching of phenyl–nitrogen bonds, the asymmetric stretching of the central C-C bond, and the bending of the ring’s C-C and C-H bonds. The latter two also redound to the band at 1304 cm^−1^. The bands at 918 cm^−1^, 760 cm^−1^, and 443 cm^−1^ are assigned to radical-ring vibration modes, along with the stretching and bending of the C-N bonds, respectively. It is evident that these spectra differ substantially from the spectrum of the initial L-GO (Figure 2b) and reflect the retention of the dyes on the surface of the adsorbent. The high-intensity bands of the dyes overlap with the main D and G peaks of L-GO. However, the 2D-overtone and D + G combinatorial bands of the initial adsorbent are clearly seen in the spectra of L-GO/TB and L-GO/MV.

**Figure 14:**
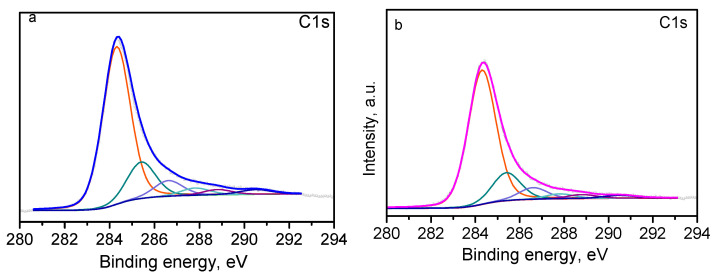
C1s XPS spectra of L-GO/TB (**a**) and L-GO/MV (**b**). Colorful lines present deconvolution, blue and magenta lines are the resulting lines.

XPS analysis was performed on the samples after dye adsorption. Figure 14 and Table 7 present the data for the binding energies related to the C1s peak of L-GO/TB and L-GO/MV.

Comparing the spectra in Figure 14 with those presented in Figure 6, it can be observed that the same components are detected in both cases. The binding energies of the peaks are identical, indicating the presence of the same functional groups on the surface of the samples. The difference is observed in the relative intensities of the peaks corresponding to different carbon bonds. Visible is the decrease in C (sp^2^) at the expense of the increase in C (sp^3^), which is due to the contribution of the methyl groups from the dyes. Some changes are observed in the content of functional groups containing oxygen as a result of the interaction of the adsorbent L-GO with the dye molecules.

The adsorption of cationic dyes on reduced graphene oxide occurs primarily through the oxygen-containing functional groups that are present on the surface. The prepared material L-GO possesses various oxygen-containing groups on its sheets, such as carboxyl (-COOH), hydroxyl (-OH), and epoxy groups. Some papers have reported that these functional groups on the surface of the reduced graphene oxide facilitate the adsorption of cationic dye molecules through electrostatic interactions [66,67]; for example, Kikkawa et al. [68] provided insights into the interactions between toluidine blue and carboxylic groups. It has also been found that GO hydrogels with a high density of carboxyl groups significantly improve the adsorption performance of various dyes, including methyl violet [69]. The adsorption of cationic dyes on reduced graphene oxide is facilitated at neutral and basic pH as the negatively charged adsorption sites on the surface are not protonated, allowing better non-electrostatic interaction with cationic dyes. The high surface area and porous texture of L-GO also contribute to the adsorption capacity, providing more accessible adsorption sites for the cationic dyes [70]. The registered change in the -OH or -COOH group content on the surface of the adsorbent after dye adsorption suggests that these functional groups are implicated in the adsorption process, which can occur through hydrogen bonding, ion exchange, or a complex mechanism [71]. The adsorption of toluidine blue also seems to involve π-π interactions between the dye molecules and the aromatic structure of the adsorbent. These considerations are also confirmed by the desorption of the dye data presented in Table 6, showing that TB has a lower desorption ability than MV (with each desorbing agent), which could indicate stronger interactions with the adsorbent. The π-π interactions for the methyl violet adsorption are not pronounced, probably due to the more distant aromatic structures in its molecule.

## 4. Conclusions

A promising graphene-based adsorbent for cationic dye removal with a high surface area and pore volume has been synthesized by a modified, environment-friendly method. The XRD, TG-DTA, FTIR, and XPS analyses have revealed that the freeze-drying process leads to a partial reduction of the prepared graphene oxide to reduced graphene oxide. This adsorbent demonstrates high adsorption capacity towards toluidine blue (265.2 mg.g^−1^) and methyl violet (200.4 mg.g^−1^) dyes from water, which is much higher than the corresponding values for most adsorbents reported. The adsorption process for both dyes follows the Langmuir model and involves physical rather than chemical interaction with the adsorbent’s surface. The adsorption of the studied cationic dyes on L-GO can be described by a combination of mechanisms, including electrostatic interactions, hydrogen bonding between the dye molecules, and the aromatic structure of reduced graphene oxide. Additionally, π-π interactions are considered to be involved in the adsorption process for toluidine blue. The findings in the present work highlight the future possibilities for enhancing the adsorption capabilities of graphene-based materials by targeted tuning to specific favorable surface oxygen-containing functional groups by appropriate preparation procedures.

## Figures and Tables

**Table 1:** Texture parameters of L-GO.

Parameter	L-GO
S, m^2^ g^−1^	734
V_t_, m^3^ g^−1^	0.52
D_av_, nm	2.9
V_mi_, m^3^ g^−1^	0.24
D_mi_, nm	1.6

**Table 2:** Kinetic parameters for TB and MV adsorption.

Dye	Pseudo-First-Order Constants			Pseudo-Second-Order Constants			Intraparticle Diffusion Constants		
	Q_e_, mg g^−1^	k_1_ min^−1^	r^2^	Q_e_ mg g^−1^	k_2_ g mg^−1^ min^−1^	r^2^	k_id1,2_ mg g^−1^ min^−1/2^	C mg g^−1^	r^2^
TB	43.58	0.024	0.7605	215.52	0.201	0.9994	23.743	43.28	0.7955
							0.225	207.03	0.8355
MV	76.56	0.021	0.8727	149.48	0.070	0.9988	16.004	6.26	0.8598
							0.173	138.42	0.9456

**Table 3:** Isotherm constants and correlation coefficients for TB and MV adsorption *.

Dye	Isotherm Model	Equation	Values	
TB	Langmuir	C_e_/Q_e_ = 1/K_L_Q_0_ + C_e_/Q_0_	Q_0_ (mg g^−1^)	265.25
			K_L_ (L mg^−1^)	0.095
			r^2^	0.9989
TB	Freundlich	ln Q_e_ = lnk_F_ + (1/n) lnC_e_	k_F_ (mg^1−n^L^n^ g^−1^)	89.00
			n (L mg^−1^)	5.52
			r^2^	0.9573
TB	Dubinin–Radushkevich	ln Qe = ln Q_m_ − βε^2^	Qm (mg g^−1^)	210.40
			E (kJ mol^−1^)	2.92
			r^2^	0.7646
MV	Langmuir	C_e_/Q_e_ = 1/K_L_Q_0_ + C_e_/Q_0_	Q_0_ (mg g^−1^)	200.40
			K_L_ (L mg^−1^)	0.024
			r^2^	0.9928
MV	Freundlich	ln Q_e_ = lnk_F_ + (1/n) lnC_e_	k_F_ (mg^1−n^L^n^ g^−1^)	36.48
			n (L mg^−1^)	3.82
			r^2^	0.9722
MV	Dubinin–Radushkevich	ln Qe = ln Q_m_ − βε^2^	Q_m_ (mg g^−1^)	145.91
			E (kJ mol^−1^)	0.35
			r^2^	0.6719

* where C_e_ is the dye concentration in the equilibrium solution (mg L^−1^); Q_e_ is the amount of dye adsorbed (mg) per unit mass of adsorbent (g); Q_0_ and Q_m_ are the maximum adsorption capacity (mg g^−1^); K_L_ and k_F_ are the Langmuir and Freundlich constants; β is the adsorption energy constant (mol^2^ J^−2^); ε is the Polanyi potential; and E is the mean adsorption energy.

**Table 4:** Comparison of the maximum adsorption capacity of L-GO with other adsorbents for the TB and MV dyes.

Adsorbent	Dye	Q_max_, mg g^−1^	References
Lemna minor	TB	26.24	[3]
Manganese/graphene-coated titaniferous sand	TB	5.32	[45]
Polyurethane foam/zinc oxide nanocomposite	TB	91.7	[46]
Magnetic Bacillus niacini nano-biosorbent	TB	82.88	[47]
Remnants of tea leaves	TB	81.3	[48]
Carboxymethylcellulose magnetic composite	TB	100.0	[49]
Activated carbon-containing porous cellulose beads	TB	123.5	[50]
Almond shell	TB	72.99	[51]
Graphene oxide/bentonite composites	TB	458.7	[52]
Freeze-dried graphene oxide	TB	265.25	Present study
Halloysite nanoclay	MV	27.85	[53]
poly(methacrylic acid-co-acrylamide)/bentonite nanocomposite	MV	137.037 149.32	[54]
Modified Saccharum bengalense	MV	7.30	[5]
AC/CoFe_2_O_4_	MV	83.90	[55]
Calcium alginate hydrogel grafted with poly (styrene-co-maleic anhydride)	MV	109.9	[56]
Acid-activated clays	MV	7.85	[57]
Kaolinite	MV	24.75	[58]
Onion skins	MV	2.23	[59]
g-C_3_N_4_-modified zeolite	MV	384.61	[60]
Freeze-dried graphene oxide	MV	200.40	Present study

**Table 5:** Desorption of dyes from L-GO.

Desorbing Agent	Desorption, %	
	TB	MV
ethanol	16	19
1M HCl–ethanol (50:50)	75	79
1M NaCl–ethanol (50:50)	88	93

**Table 6:** Texture parameters of L-GO/TB and L-GO/MV.

Parameter	L-GO/TB	L-GO/MV
S, m^2^ g^−1^	470	411
V_t_, m^3^ g^−1^	0.39	0.33
D_av_, nm	3.3	3.2
V_mi_, m^3^ g^−1^	0.12	0.10
D_mi_, nm	1.9	1.4

**Table 7:** Surface composition and peak deconvolution of C1s of L-GO/TB and L-GO/MV.

	Concentration				C1s deconvolution					
Sample	C at.%	O at.%	N at.%	S at.%	C (sp^2^) %	C (sp^3^) %	-C-O %	-C=O %	-COOH %	π-π %
L-GO	93.8	6.2	-	-	76.5	12.0	5.3	2.0	2.4	1.8
L-GO/TB	90.3	6.9	1.9	0.9	68.7	16.4	7.0	3.3	2.3	2.3
L-GO/MV	92.4	6.2	1.4	-	72.1	15.2	6.3	2.6	2.1	1.7

## Data Availability

The original contributions presented in the study are included in this article/the Supplementary Materials. Further inquiries can be directed to the corresponding author.

## Supplementary Materials

The following supporting information can be downloaded at https://www.mdpi.com/article/10.3390/ma18040853/s1: Figure S1: Calibration curve of methyl violet, Figure S2: Calibration curve of toluidine blue.
