# Supplementary material for: A Highly Efficient Graphene-Based Material for the Removal of Cationic Dyes from Aqueous Solutions
Source: Materials (Basel). 2025 Feb 15;18(4):853. doi: 10.3390/ma18040853 (PMC11857711; doi:10.3390/ma18040853)
Supplement: Supplementary file 1 [file materials-18-00853-s001.zip › materials-3446406-supplementary.pdf]

Supplementary Materials

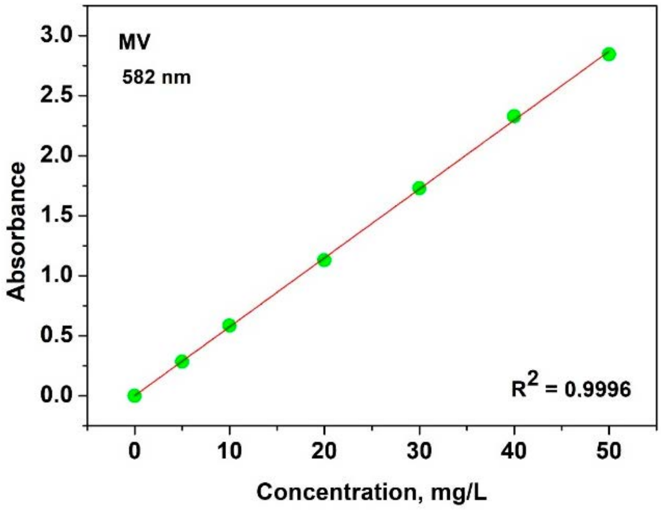

Figure S1. Calibration curve of methyl violet.

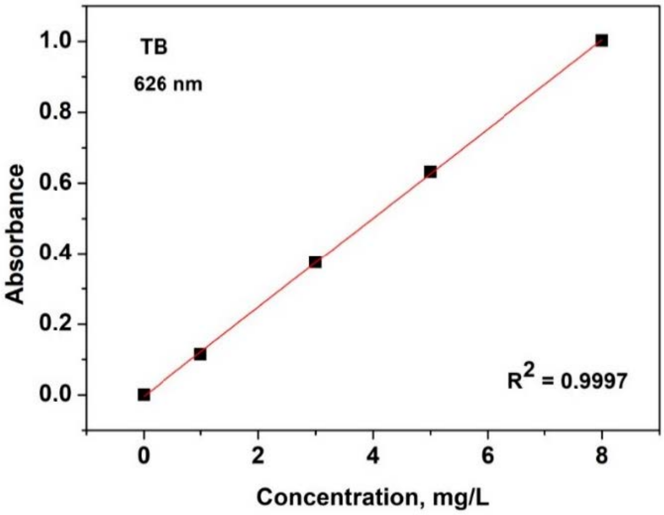

Figure S2. Calibration curve of toluidine blue.
